# High-Level Teleoperation System for Aerial Exploration of Indoor Environments

**DOI:** 10.3389/frobt.2019.00095

**Published:** 2019-10-23

**Authors:** Werner Alexander Isop, Christoph Gebhardt, Tobias Nägeli, Friedrich Fraundorfer, Otmar Hilliges, Dieter Schmalstieg

**Affiliations:** ^1^Institute of Computer Graphics and Vision, Graz University of Technology, Graz, Austria; ^2^Advanced Interactive Technologies Lab, ETH Zürich, Zurich, Switzerland; ^3^VRVis Research Center, Vienna, Austria

**Keywords:** teleoperation systems, telerobotics, interactive scene topology, scene abstraction, indoor exploration tasks, search and rescue, unmanned aerial vehicles - UAV

## Abstract

Exploration of challenging indoor environments is a demanding task. While automation with aerial robots seems a promising solution, fully autonomous systems still struggle with high-level cognitive tasks and intuitive decision making. To facilitate automation, we introduce a novel teleoperation system with an aerial telerobot that is capable of handling all demanding low-level tasks. Motivated by the typical structure of indoor environments, the system creates an interactive scene topology in real-time that reduces scene details and supports affordances. Thus, difficult high-level tasks can be effectively supervised by a human operator. To elaborate on the effectiveness of our system during a real-world exploration mission, we conducted a user study. Despite being limited by real-world constraints, results indicate that our system better supports operators with indoor exploration, compared to a baseline system with traditional joystick control.

## 1. Introduction

Teleoperation of small-sized aerial robots in indoor environments is important for applications like search-and-rescue or exploration missions. A recurring problem in such applications is lack of situation awareness and consequently decreasing overall task performance (Burke and Murphy, 2004; Stubbs et al., 2007).

One important aspect is that with an increasing amount of scene details, operators struggle to comprehend the visualized information of the teleoperation system (Atherton and Goodrich, 2009). While it is required that the system presents the information in a way that does not overwhelm the operator, also the levels of autonomy (LOA) play a crucial role. Increasing autonomy of the system can improve operators task performance by reducing their mental load. The goal is to free up the operators to be engaged in other important high-level tasks (Goodrich et al., 2007), such as navigation or identification of victims or hazards. However, related work has shown that true full autonomy is still hard to accomplish for complex missions (MahmoudZadeh et al., 2018). This emphasizes difficulty of an optimal level of autonomy for a teleoperation system. As a tradeoff, approaches were introduced in which operators can explicitly adjust the autonomy of the system to the desired level (Bruemmer et al., 2002; Muszynski et al., 2012). Unfortunately, such approaches typically require a handover to low-level demanding tasks (Reese, 2016). While trading off task automation and manual control is task-specific and remains non-trivial to date, our system, on one hand, suggests to automate all low-level tasks. On the other hand, high-level tasks can be accessed via an interactive scene with reduced details. Yet, the question remains how such a system effects aerial exploration missions in a real-world setting.

**Figure 1:**
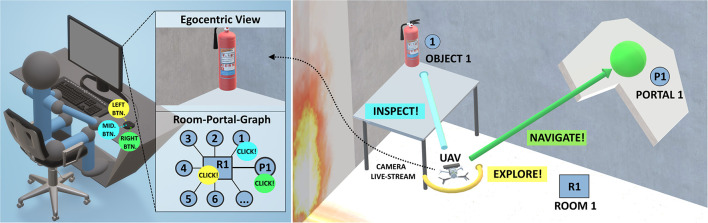
High-Level teleoperation system. (**Left**) The room-portal graph displays an interactive topological view of an indoor environment, created in real-time, to facilitate automation. (**Right**) Conceptual illustration of the aerial telerobot, implemented as unmanned aerial vehicle (UAV), and its according high-level tasks.

To this aim, we introduce a fully working teleoperation system. The system uses a small-sized aerial telerobot to perform the challenging task of indoor exploration (Figure 1). In particular, our system is capable of: (i) indoor navigation in the presence of narrow spaces and wind-turbulence without collision; (ii) automated repetitive exploration and navigation of structured spaces; (iii) detection and navigation of constrained spaces, like narrow gateways or windows, without collision; (iv) and detection of objects of interest (OOIs), like victims or fire extinguishers. To relieve the operator, the system automates all low-level mission-critical tasks. However, we allow the operator to override non-mission-critical, high-level objectives. This results in a design where the system usually runs at the highest LOA (*highest automation*), but can be effectively supervised at collaborative level (*high automation*) if necessary (see Table 1)^1^.

**Table 1:** Relation between the ALFUS and operating the UAV of our teleoperation system.

**ALFUS**	**Level definitions**	**Operating the UAV**
[10] Approaching 0% HRI	Highest level of automation, high complexity, all missions, extreme environment	Maximum automation level. Full autonomous exploration of the environment including object detection. The user can still improve task performance by switching to the collaborative levels (e.g., navigation of one preferred portal over the other or inspection of an OOI for verification).
[7–9] Low-level HRI	Collaborative level of automation, high complexity missions, difficult environments	High automation level involving non-mission-critical tasks. Functioning of repetitive low-level tasks is guaranteed (collision-free navigation). The operator can switch to this level, to supervise the system if it fails with complex tasks on highest level. Minimum level that the operator can access in the RPG condition.
[4–6] Medium-level HRI	Medium level of automation and complexity of missions, multi-functional missions, moderate environment	No operator access at this level for RPG condition.
[1–3] High-level HRI	Low level of automation, low level tactical behavior, simple environments	No operator access at this level for RPG condition. We use that range of levels in the JOY condition.
[0] 100% HRI	Lowest level of automation, full manual remote control by the operator	No operator access at this level for RPG condition.

**Figure 2:**
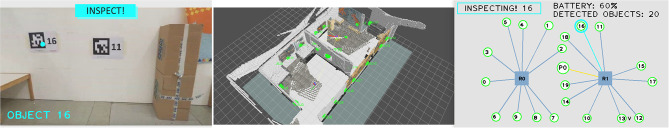
Implementation of the RPG as part of our high-level teleoperation system. It is presented during an inspection task, after full exploration of two connected rooms. (**Left**) Egocentric virtual live view from the on-board camera of the UAV, highlighting an inspected object. (**Middle**) Exocentric virtual 3D view of the reconstructed scene. (**Right**) Interactive, topological RPG of the same scene, with two rooms (represented as squares), detected objects including a portal. Objects are shown as round labels with leader lines. Inspected objects are highlighted.

The operator supervises the teleoperation system using a multi-view GUI which consists of a traditional egocentric scene view, an exocentric scene view, and a complementary topological graph view of the scene, called the room-portal graph (RPG) (see Figure 2). The RPG represents the scene as a subdivision of rooms connected by portals, creating a clearly distinguishable spatial structure of a typical indoor environment. The RPG reduces scene details and allows fast comprehension of important scene structure and objects during an exploration mission. It is interactive and lets the operator improve time-performance and resolve system failures, for example false detection of OOIs.

To understand the task effectiveness of our teleoperation system in a real-world setting, we conducted a user study. Participants accomplished an exploration mission using our proposed system and, in comparison, using a baseline system with traditional joystick control. While results indicate increased task performance and comfort with the outcome of our experiments, our findings provide evidence that our system can better support operators with challenging aerial indoor exploration missions.

In summary, we contribute:(i) a fully working teleoperation system for aerial indoor exploration missions, including a real-time interactive scene topology that enables supervisory control of high-level tasks, and (ii) the empirical evidence that such a system effectively supports human operators during realistic exploration missions. In the remainder of the paper, we provide an extensive overview on related work, including a brief history of teleoperation and current state-of-the-art systems. We present details of design rationals and implementation of our system, as well as limitations, followed by a report on experimental design and results of our user study. We conclude our paper with interesting directions for future work.

## 2. Related Work

The research conducted in the area of robotic teleoperation is extensive and was explored starting from the mid of the twentieth century. The research is highly interdisciplinary and addresses a rich variety of aspects in human-robot-interaction, mobile robotics and visualization. The purpose of this section is to help the reader to understand the complexity of the field and to provide an overview of state-of-the-art systems. It starts with a short historical summary of teleoperation. It continues with a broad range of related work regarding telerobotics and ends with addressing specific work on high-level indoor exploration with aerial teleoperation systems. Thus, we provide an extensive literature survey and discussion about origin and history of robotic teleoperation, its motivation, typical use cases and changing demands to systems over time. Finally, it adds a discussion about limitations and potential future work to the individual subsections. Ultimately, our goal is to help the reader to better understand the motivation of our system and its novelty in the presented form.

### 2.1. History of Teleoperation - From the Poking of Fire to Telerobotics in Space

An extensive survey by Lichiardopol (2007) suggests that the first form of teleoperation was the poking of fire in the early age of human mankind. By utilizing the stick to set fire, the human was actually teleoperating (or telemanipulating) the fire place. A million years later, in the early 1900s, for the first time teleoperation appeared for piloting unmanned aircrafts (Fong and Thorpe, 2001). Related work continues in the mid of the past century, but in the context of remote handling hazardous materials. According to an extensive summary of Vertut and Coiffet (1986), R. Goertz worked on a pantograph as telemanipulation device for radioactive materials inside a nuclear reactor. The obvious purpose was to enable safe handling of the otherwise dangerous materials by human operators. In the following decades, the need for robotic teleoperation significantly increased, also highlighted as part of a comprehensive survey of A. D. Alexander (1972). In his work, A. D. Alexander (1972) subsumes the terms teloperation and telemanipulation under “telemation” and investigates on which use cases in the civil sector development on teleoperation systems would have greatest impact. Amongst nuclear reactor fuel handling, mining, oceanographic and medical teleoperation systems, additionally a strong need for teleoperation was observed in aeronautics and space operations (Corliss and Johnson, 1967; Alexander, 1973). At this point, the original purpose of robotic teleoperation becomes apparent, which is to enable a human operator to work at a (larger) distance in hazardous environments. According to Lichiardopol (2007), this requires the following essential definitions:

- **(Autonomous) Teleoperator:** The automatically operated machine that replaces human effort, though it may not resemble human beings in appearance or perform functions in a humanlike manner.
- **Operator:** A human operator is the person who monitors the operated machine and takes control of the required actions.
- **Teleoperation:** The task or activity itself to “operate a vehicle or a system over a distance”(Fong and Thorpe, 2001).

More recent work on teleoperation (Cui et al., 2003) gives a clearer overview of the required main components, data-flow, and interfaces. These are in particular:

- Remote System (**teleoperator or telerobot**): a machine or robot that enables a human operator to move about, sense and mechanically manipulate objects at a distance.
- Human Machine Interface (**human-robot interface**): a software framework, including a graphical user interface (GUI) control software, and interface software to process sensory data.
- Manual Control Unit (**input device**): an input device for manual control inputs.

By definition, the human operator manually interacts with the teleoperation system via the input device, whereas the telerobot senses and performs actions in the far distant environment.

### 2.2. Telerobots—Reducing Human Effort

According to Cui et al. (2003), telerobots are a subclass of teleoperators. They are typically implemented as mobile robots that accept instructions from a human operator at a distance. Moreover, they perform live actions at a distant environment through the use of sensors or other control mechanisms. Usually they have sensors and/or effectors for manipulation and/or mobility, and moreover provide mechanisms for the human operator to communicate with both. Due to the rich variety of applications and remote environments, related work addressed various types and sizes of mobile robots during the past decades. Such are ranging from space exploration-, mining-, medical-, and underwater-applications (A. Alexander, 1972; Sheridan and Verplank, 1978) in the early years of modern teleoperation, to humanoid telepresence robots with increasing popularity in the early 2000s (Lichiardopol, 2007) and more recent telerobots for bomb defusal (Technology, 2014). Amongst others, important types of telerobots include stationary robots like arm manipulators (Murray, 2017; Rakita et al., 2018), underwater robots (Costa et al., 2018), ground based search and rescue robots (Stepanova et al., 2017), humanoid robots for telepresence (Cortellessa et al., 2018) and aerial telerobots for surveillance (Jha, 2016) or exploration and mapping (Papachristos et al., 2017).

In particular, small sized unmanned aerial vehicles (UAVs) or micro aerial vehicles (MAVs) soared popularity in the past decades (Cai et al., 2014). While their small size and low weight make them attractive for research and education (Wu et al., 2018), they are also extensively used outdoors for industrial purposes. Typical use cases involve agriculture (Tripicchio et al., 2015), medical transportation (Scalea et al., 2019) and moreover search and rescue or exploration missions (Silvagni et al., 2019). However, such use cases put different demands on the UAV, compared to indoor flights. In outdoor environments spatial constraints play a subsidiary role, and, very often, GPS-based localization and navigation is possible. In contrast, exploration of indoor environments requires small UAV dimensions to avoid problems with reaching narrow passages. Also, limitations apply to onboard computational power and flight times. Furthermore, low-thrust propulsion is important, since turbulences can strongly affect stability and overall flight performance of the UAV. State of the art off-the-shelf UAVs which could be potentially used for such purposes are the DJI Mavic or the Intel Aero platform. However, on one hand, such solutions are more bulky and heavy and are not easily customizable (software interfaces, sensors). On the other hand, smaller and more lightweight solutions (Kushleyev et al., 2013; Loianno et al., 2016) are more limited regarding payload and flight times. Very recent UAV designs with similar all-up-weight and dimensions (Sanket et al., 2018; Falanga et al., 2019) also discuss navigation and exploration of portals. However, they either provide shorter hover flight times or do not carry Kinect-like RGB-D sensors. Such are used for creating dense mapping data (Henry et al., 2012), which provides benefits for indoor exploration. In general, to achieve an optimal UAV design for aerial exploration and online mapping of indoor environments still remains difficult. A potential future design, also reflecting the room for improvement of our presented UAV, may include the following challenging specifications if combined in one system:

- **Small-scale dimension** with tip-to-tip diameter under 100 mm (Giernacki et al., 2017) (approx. size of the palm of a humans hand) which would enable it to reach very narrow passages and make it easy to hold and carry.
- **Low all-up-weight** below 100 g (Kushleyev et al., 2013) to make transportation easier and reduce produced thrust and turbulences during flight.
- **Low-weight sensors** including Kinect-like or omnidirectional vision sensors.
- **Powerful computing units** (e.g., Tegra K1; Kyristsis et al., 2016), including GPUs to execute all tasks which are important for exploration of indoor environments onboard (robust localization, exploration, navigation, motion planning, online mapping, and object recognition).
- **Increased flight times** of more than 30 min, which is typical for state-of-the-art commercial UAVs in this size- and weight-category (Robotics, 2019), or extendable flight times by autonomous wireless recharging technology (Junaid et al., 2016; Al-Obaidi et al., 2019).

### 2.3. Remote Connection - Coping With the Issues of Time Delay

Although the remote connection between telerobot and human-robot interface is not listed as an individual component by Cui et al. (2003), it has major impact on the overall task performance. According to a survey of Sheridan (1993), time delay is a “serious problem for teleoperation.” Issues with time delays were for the first time addressed in the early 1960's by Adams (1961), whereas later work by Ferrell (1965) found that human operators can cope with time delays by using a simple “move and wait strategy.” His experiments also showed that task performance is linearly dependent and predictable on the time-delay during teleoperation. Remarkably, Held et al. (1966) found that sensory-motor adaptation is essentially impossible for delays as small as 0.3 s, and that human operators dissociate the teleoperator movements from those of their own in the presence of such delays (Held and Durlach, 1991). Especially if direct control methods are used, this could lead to a non-optimal task performance and (Sheridan, 1992) explicitly states that direct control in the presence of delay (transmission or otherwise) is tedious, fatiguing, and error prone. Since our teleoperation system includes a wireless remote connection with potential higher time-delays (>0.3 s), this also affected our design decisions about the control approach (section3.2.1). More recent related work discusses impact of time delays during teleoperation of small-sized UAVs (Riestock et al., 2017a,b), whereas they elaborate on effects of limited bandwidth on the GUI. They compare operators performance during collision avoidance tasks and use traditional egocentric live camera views and grid-map representations of the scene. Their results indicate that the operator performance suffered less under a change of communication quality using grid-maps, compared to the egocentric live camera views. Consequently this was considered in the design of our GUI (section 3.2.2).

### 2.4. Human-Robot Interfaces—Facilitating Control and Cognition

Based on the summary of Cui et al. (2003), the human-robot interface processes data of sensors and actuators of the telerobot for control, typically at different LOA. It further visualizes information about the remote system and the remote scene with a GUI. Finally, it is responsible for processing the operator's manual inputs of the input device.

#### 2.4.1. Levels of Automation and Approaches for Control

The LOA of a teleoperation system is important, since it could have great impact on the overall design of the teloperation system (Save et al., 2012; Endsley, 2018). Moreover, the LOA greatly effects the operators overall task performance during teleoperation (Materna et al., 2017). Subsequently, also a variety of taxonomies for LOA were introduced for robotic teleoperation. While the idea of LOA was introduced by Sheridan and Verplank (1978) in the late 1970s for underwater teleoperation applications, more recent work broadened this concept (Endsley, 1999; Parasuraman et al., 2000). Depending on the application, related work discusses various models with more or less fine grained LOA for flight traffic control (Wickens et al., 1998), manufacturing (Frohm et al., 2008) and, most recently, autonomous driving (SAE, 2014). Most interesting four our work is a LOA-taxonomy specifically designed for unmanned systems. Introduced by Huang et al. (2005a,b), it was successfully adapted for indoor exploration missions by Valero-Gomez et al. (2011).

On one hand, low LOA typically imply that the human operator has direct *manual control* over the telerobot and can also directly access detailed sensory data (Sheridan, 1992). However, the operator must be also able to process and interpret this data. During challenging tasks under time constraints, this could overwhelm the operator and lead to decreased task performance or even mission failure. In contrast, so-called “fully automated systems" without any control of an operator are still hard to put into practice. At least, their overall performance can be still significantly improved if they are teamed with a human (Johnson and Vera, 2019). In order, also the design of our human-robot interface is motivated by the capabilities and moreover limitations of the telerobot. The presented GUI builds on top, enabling the human to supervise with difficult high-level tasks. Automation at higher levels means that the telerobot is able to accomplish certain low-level tasks independently and could relieve the operator based on *supervisory control* (Sheridan, 1992). If the telerobot fails, on demand switching the system to lower LOA could be helpful (*adjustable or sliding autonomy*), whereas an extensive survey is presented by Mostafa et al. (2019). Bruemmer et al. (2005), Leeper et al. (2012), Chen et al. (2013), and Muszynski et al. (2012) propose teleoperation systems with different LOA for control of ground-based robots from classical egocentric and exocentric views. These works consistently report on improved operator performance with increasing autonomy. Extensive research has been conducted concerning the concept of switching between LOA. Valero-Gomez et al. (2011) suggest two autonomy models to interact with robot teams during exploration missions and enable low-level operation on demand. Fong et al. (2003) explored semi-autonomous systems that query users for input, in case of uncertain decision. Both papers suggest that system failures should be handled manually by the operator. However, their design focuses on ground based navigation or grasping. They also do not provide a minimum LOA to the operator, avoiding mission-critical tasks during flight missions. To cope with the issues of direct control, Gebhardt et al. (2016) and Nägeli et al. (2017a,b) suggest optimized planning of constrained quadrotor paths. They also avoid passing low-level tasks to the operator and instead introduce indirect, high-level flight goals. They allow inexperienced operators to control the UAV without deeper knowledge of the underlying methods for quadrotor control or the target domain. An important prerequisite for such ease of use is that the UAV can move along a collision-free path. More recent work combining supervisory control and adjustable autonomy is presented by Lin and Goodrich (2015), Lan et al. (2017), and Szafir et al. (2017). Remarkable limitations with all supervisory control approaches are the *lumberjack effect* (Onnasch et al., 2014) and the *automation conundrum* (Endsley, 2017). These effects summarize a tradeoff between high LOA improving task performance and problems with sudden passing of low-level tasks if problems occur. Moreover, a general concept that provides an optimum LOA for all applications and tasks seems impossible today and will remain difficult in the future. Such limitations must be considered in the design of the teleoperation system. Consequently, our system avoids sudden passing of low-level functions to the operator and only allows for overriding functions at high LOA (supervisory control). In contrast to related work, the LOA design of our work considers challenging tasks that occur during aerial indoor exploration missions. Design details can be found in section 3.2.

#### 2.4.2. Graphical User Interfaces

Various types of GUIs, with different combination of scene views, have been investigated to improve task efficiency for teleoperation of small-sized UAVs. Examples range from interfaces with traditional egocentric live camera views (Cho et al., 2017), combined with direct joystick based control, to fully immersive interfaces utilizing the operators upper body for control (Rognon et al., 2018).

As an alternative to UAV navigation from egocentric views, direct commands can be issued in an adaptive exocentric perspective (Saakes et al., 2013; Thomason et al., 2017, 2019) or from a 3D map view (Materna et al., 2017). The exocentric view can improve the operator's understanding of the environment and further increase safety and task performance. Additionally, concepts of switching between ego- and exocentric views is discussed by Baudisch et al. (2002), whereas Ferland et al. (2009) suggest to switch between egocentric and exocentric views for robot teleoperation. Following the overview- and detail paradigm, their overall goal is to improve task performance by providing information details on demand. According to Gebhardt et al. (2016) and Nägeli et al. (2017a), pure exocentric planning views can be beneficial for applications such as robotic light painting, drone-racing and aerial cinematography. Also the work of Lan et al. (2017) combines exocentric scene views with a high-level GUI for photo taking with a semi-autonomous UAV. However, these applications do not generate an interactive scene topology from 3D data. They either require that a 3D map is already pre-generated or use mapping for localization and path planning only. Importantly, they do not consider challenging tasks that occur during indoor exploration missions, like flight through narrow passages. In contrast, the design of our system focuses on flight missions in challenging indoor environments. Most importantly, we generate an interactive scene topology in real-time and thus facilitate automation.

Relying solely on an exocentric 3D map can lead to problems. For example, Chellali and Baizid (2011) state that on one hand the third dimension is an additional degree of freedom that helps to add constraints and information to disambiguate location of objects. On the other hand, they report on significantly decreased task performance when localizing objects in 3D maps, compared to localization in 2D. They suggest that the additional dimension provided within 3D maps leads to a greater space to explore and thus the operator needs more time. This tradeoff was also considered in the design of our GUI, which is outlined in section 3.2.2.

**Figure 3:**
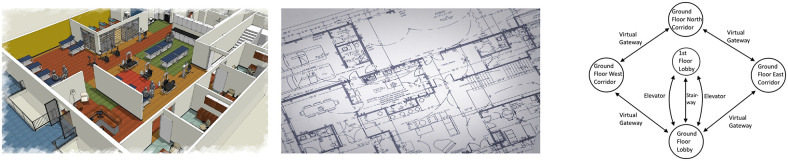
Example map views of complex office environments with gradual loss of details. (**Left**) Full 3D map view. (**Middle**) Floorplan in 2D. (**Right**) Topological view (Kun et al., 2017).

By even more reducing scene details, abstract topological views prevent the operator from being overwhelmed and typically rely on 2D or 3D data (Richtsfeld et al., 2012; Yang and Worboys, 2015; Wang et al., 2018). Bormann et al. (2016) and Ambrus et al. (2017) introduce segmentation of rooms in indoor environments. The goal of their work is to provide the segmented data to mobile robots for autonomous navigation. In contrast, Kortenkamp (1993), Choset and Nagatani (2001), Schröter et al. (2003), Vasudevan et al. (2007), and Angeli et al. (2008) represent the environmental understanding of a mobile robot in a way that facilitates human comprehension. They suggest a topological representation of places visualized as object graphs. The visualization of the environment is hierarchical, and strongly motivates usage for navigation. However, highlighting OOIs in real-time during flight missions is not investigated. Yang and Worboys (2015) also supports structuring of indoor spaces into rooms and portals from offline generated data. Kun et al. (2017) report on ontology-based navigation as part of an indoor positioning framework, introducing basic categories of abstract 2D objects (right Figure 3). All these approaches strongly support design of an abstract comprehensive representation of the scene to compute interactive navigation graphs for an indoor space (section 3.2.2). However, none of these authors evaluate real-time generation of an interactive scene topology as part of a teleoperation system for aerial indoor exploration under real-world constraints.

### 2.5. Input Devices—Enabling Manual Control

Extensive research on teleoperation of small sized aerial robots with various types of input-devices and according interactions, has been conducted in the past decade. Despite the popularity of novel interaction paradigms, like hand-gesture (Yu et al., 2017; Duan et al., 2018), body-gesture (Rognon et al., 2018), gaze (Yu et al., 2014; Erat et al., 2018; Yuan et al., 2019), and language (Huang et al., 2010, 2019), more recent work still focuses on aspects of traditional joystick based teleoperation of small-sized UAVs, for example avoiding collisions during navigation (Cho et al., 2017). Sanders et al. (2018) report that operators still prefer joystick control over indirect gaze-based steering, whereas findings of Herrmann and Schmidt (2018) indicate that a traditional input device is more efficient than their extensive and carefully designed system based on natural interactions. In conclusion, if task efficiency is preferred over user experience (fun to use, increasing enjoyment during teleoperation) traditional input devices are still hard to outperform. Remarkably, joystick controls can be still considered as state-of-the-art input device and are commonly chosen as baseline for performance evaluations. As a consequence, also our work aimed for selecting a traditional input device and according interaction design which is able to compete against conventional joystick controls. Experimental results are detailed in section 6.1. Other important aspects were required pre-training, complexity and cost-effectiveness. Details of the according design rationales can be found in section 3.3.

### 2.6. High-Level Teleoperation Systems for Exploration of Indoor Environments

Very recent work on fully working teleoperation systems for indoor environments is discussed by Valner et al. (2018). In their work, they introduce high-level interaction methods based on gesture and language, but for ground-based robots. While they also suggest seamless switching between navigation, inspection and manipulation tasks, they use traditional egocentric 2D views and a 3D map to improve task performance. Recent work on fully working teleoperation systems, but with aerial telerobots is discussed by Hedayati et al. (2018), Huang et al. (2019), Paterson et al. (2019) and Walker et al. (2019). All systems use state-of-the-art AR, MR, or VR input devices, whereas they also design high-level interactions for their human-robot interface. Their overall goal is to improve task-efficiency when commanding aerial telerobots in indoor environments. Remarkably, they all compare their teleoperation systems against baseline systems (using traditional joystick or keyboard controls) and their independent variable in the study corresponds to what type of teleoperation interface the participants used. However, their systems are based on natural gaze or language commands and do not refer to an interactive 2D scene topology created in real-time. Further, they do not consider aerial exploration missions in challenging indoor environments where simple and robust input devices can be beneficial to improve task performance. Related work, which might be closest to ours, is presented by Szafir et al. (2017). The work presents three prototypes with different methods to control an aerial telerobot. Interestingly, they also make use of an abstract floor-plan representation of the scene. However, this plan is static and not autonomously created in real-time. Although related work already proposed abstract topological views for the control of teleoperation systems, to our understanding we are the first who introduce a fully working system that refers to an interactive scene topology, created in real-time during flight. This raises the interesting question, if the performance of our teleoperation system is also preserved when put into practice. Compared to a variety of related teleoperation systems with similar mission complexity (Cho et al., 2017; Riestock et al., 2017b; Thomason et al., 2017), we evaluate the performance of our system with a user study under real-world constraints (section 6.1).

## 3. System Design Rationales

**Figure 4:**
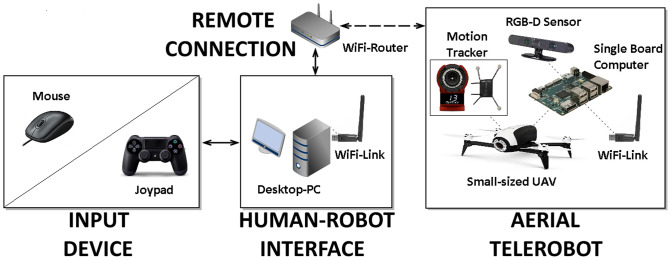
Overview of the main components of the teleoperation system design: The aerial telerobot which is a small-sized UAV and localized by a motion tracker (Optitrack, 2019). A remote connection between the telerobot and the human-robot interface which runs on a desktop PC. The input devices used for manual control of our teleoperation system.

The design of our teleoperation system is governed by the needs of aerial exploration. It focuses on exploration of civil buildings with constrained indoor spaces and repeating room geometry. Example representations of an office building are shown in left and middle Figure 3 (3D map and 2D floorplan). Typically, an exploration mission would require to navigate inside the building and detect OOIs (fire extinguishers or trapped victims). For such applications teleoperation systems can be helpful, if disaster relief forces are not able to reach inside such buildings, and assessment of the situation is required (Lichiardopol, 2007). Our teleoperation system uses the same main components as state-of-the-art systems (Figure 4):

- Aerial Telerobot: Our telerobot is a small-sized UAV, holding various sensors (cameras and inertial sensors) and actuators to perform the challenging task of aerial indoor exploration. Additionally it is equipped with an onboard computer to transfer sensor and actuator data to the human robot interface via a wireless remote connection.
- Human Robot Interface: Our human robot interface includes all software components for processing the sensory data and flight-control of the telerobot. Further it holds the interactive scene topology (RPG) which is enabled by the underlying system components (section 4.3).
- Input Device: The design of our system considers a simple and cost-effective input device sending manual high-level commands to the human-robot interface.

### 3.1. Teleoperated Aerial Exploration of Indoor Environments

**Figure 5:**
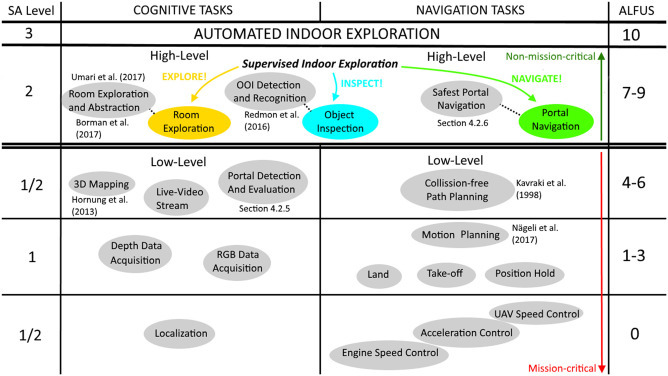
Overview of high-level and according low-level sub-tasks that have to be solved by our teleoperation system. Noteworthy is the separation between cognitive- and navigation-tasks, definition of mission-critical tasks, levels of situation awareness (Burke and Murphy, 2004), relation to the ALFUS (Huang et al., 2005b), and the recovery behaviors, triggered by high-level interactions of the operator (Explore!, Inspect!, and Navigate!).

Indoor space is typically limited and room exploration may require passing through narrow passages or so called portals, which can be hallways or windows. As a consequence, for our teleoperation system we designed a highly mobile and rather small sized UAV as telerobot. While important aspects are mentioned in section 2.2, the design of our telerobot focuses on core functionalities which are vital for indoor exploration. On a higher task-level, our telerobot provides functions for **room exploration**, **object inspection**, and **navigation of portals**. However, such high-level tasks entail a variety of low-level functionalities with increased complexity (Figure 5). Also, it is important to distinguish between **mission-critical tasks** and **non-mission-critical tasks**, whereas mission-critical tasks have to be solved by the teleoperation system under all circumstances and at all time. If the system fails with a mission-critical task this could lead to serious damage of the telerobot and potentially end the overall mission. For our system design we define the following low-level mission-critical tasks which are vital for indoor exploration:

- Localization: The telerobot has to be able to localize itself against the environment at all time. A failure in self-localization would typically result in that the telerobot collides with its environment.
- Landing/Take-off: Based on a robust localization and proper control of speed and acceleration, the telerobot provides assistive features like take-off and landing.
- Hold Position: Due to the turbulences that occur in the indoor environment, our design has to consider methods for stabilizing the telerobot while in-air and rejecting disturbances. Disturbances can occur due to flying close to obstacles or passing through portals.
- Collision-free Path Planning: Path- and motion-planning ensures collision-free navigation inside the indoor environment. It is vital if navigation between objects is required (waypoint-based navigation).
- Live-Video Stream And 3D Mapping: It is based on a robust acquisition of sensory data, whereas abstraction into a topology requires the 3D data. Since 3D Mapping also provides minimum understanding of the remote scene to the human operator, these tasks must not fail.
- Portal Detection And Evaluation: We detect portals by analyzing single depth images, in which we recognize the contour of the portal in 3D and estimate size (minimum diameter) and the normal vector of the contour at the geometric center. Once a potential portal is detected, it must be evaluated correctly.

On top of the low-level tasks, we introduce high-level non-mission-critical tasks. These are difficult cognitive tasks, where a human operator can still improve overall performance. The high-level tasks can be summarized to one “automated indoor exploration” task, autonomously executed by the system at highest LOA. In particular, the system uses an automated search strategy to explore one single room, identifies objects and portals on the fly and is able to navigate the safest portal. Implementation details are given in section 4. Non-mission-critical tasks are considered as the following:

- Room Exploration and Abstraction: Room exploration is based on a state-of-the-art rapidly exploring random trees (RRT) exploration algorithm. On lower level this requires collision-free navigation. In parallel the system has to tackle the challenging tasks of detecting portals for navigation and objects of importance (OOIs). Once a full room is explored it is abstracted into a node and added to the scenes topology.
- Object Detection And Recognition: For object detection our system design aims for using state-of-the-art real-time detection algorithm.
- Safest Portal Navigation: After room exploration the system navigates the safest detected portal.

However, if the system fails with one of the high-level tasks, the operator can intervene by commanding high-level recovery behaviors in the GUI of the human-robot interface (Figure 1). In detail the operator can: trigger a simplistic but robust search strategy (**Explore!**), select a preferred portal over the other (**Navigate!**) or correct for object detection by close inspecting the object and/or registering the object manually (**Inspect!**). Noteworthy is that our Inspect! command is motivated by the overview and detail paradigm, also used in the work of Seo et al. (2017) to improve effectiveness of teleoperation.

### 3.2. Human-Robot Interface

Our human-robot interface is designed to support the human operator during teleoperation. Core design aspects are typical essential tasks during aerial indoor exploration, limitations of the telerobot and usage of an untethered remote connection.

#### 3.2.1. Levels of Automation and Approaches for Control

Our proposed scenario for aerial indoor exploration involves rather complex tasks, like object recognition and path planning. Such tasks have to be executed at the same time and involve mission-critical tasks like collision-free navigation. Due to the complexity of the tasks, the design of our system assumes that true full autonomy is not feasible. For our scenario a human operator is necessary to support the system with complex cognitive tasks on higher level. However, these tasks are non-mission-critical. The purpose is to avoid the lumberjack-effect and avoid sudden passing of control to the operator. If tasks fail on higher level, the telerobot is not damaged and able to continue with the overall mission. As a consequence, in accordance to related work (Valero-Gomez et al., 2011), we design a supervisory control approach for our system which adapts the ALFUS framework (Huang et al., 2005b). Details about task definitions, high-level interactions to supervise the system with recovery behaviors and relation to LOA are presented in Figure 5 and Table 1. Importantly, hazardous regions in challenging indoor environments require the usage of an untethered remote connection. Consequently, potential sudden network dropouts and time delays during control strongly motivate supervisory control.

#### 3.2.2. Graphical User Interface

The user interface is one vital design aspect of our full high-level teleoperation system. Moreover, its design is based on the complex interplay with the underlying system components, whereas the overall goal is to improve teleoperation during aerial exploration missions. Yanco et al. (2004) summarizes core design aspects to improve overall task performance which are (1) using a map; (2) fusing sensor information; (3) minimizing the use of multiple windows; and (4) providing more spatial information to the operator. In addition, Nielsen et al. (2007) discusses several window layouts in a standard paradigm. Besides of the rich variety of designs found in related work, a very common window layout is placement of exocentric map views on the bottom half of the screen whereas egocentric live camera views are placed on top.

The design of the GUI is also based on a standard layout, whereas we keep all view windows at equal size. It includes a traditional egocentric live view on top and a 3D map view on the bottom half of the screen. The purpose is to provide a minimum of spatial understanding to the operator. For the 3D view we use grid-map representations as they are a more robust in the presence of network delays and sudden dropouts (Riestock et al., 2017b). We place the view of the interactive scene topology (RPG) side by side to the traditional views to avoid occlusions or switching. The RPG is motivated by exploration of structured human environments, which can have complex and repetitive geometry (e.g., office buildings). While the structure of such environments motivates a topological representation of the environment, related work (section 2.4.2) clearly supports the use for navigating robots. Other motivational aspects are extensively discussed by Johnson (2018). Amongst other benefits, the work states that a topological representation is suitable for telerobots which have to navigate reliably from one place to another without the need of building an accurate map. While this is not valid during exploration of the environment, clear benefits occur for repeated navigation from one object to another after exploration. Johnson (2018) also points out that a topological view supports affordances (opportunities for interactions) and poses a human-like representation of the environment. Based on the concept of an ecological interface (Nielsen et al., 2007), we designed visualization of objects that support affordances, but do not overwhelm the operator (Atherton and Goodrich, 2009). Consequently, we define general OOIs, which are detected during the exploration mission and highlighted in the topological scene view. Based on these considerations and avoiding to overwhelm the operator with too rich scene details in the traditional views (left and middle Figure 3), our design leads to the RPG which poses an interactive topological 2D map of the indoor environment. Implementation details can be found in section 4.2.

### 3.3. Input Device

The design of our high-level teleoperation system includes a topological scene view which is represented in 2D. Because the topology supports affordances, we make OOIs explicit for interaction in the RPG, during flight. Motivated by the 2D representation and also considering the design aspects discussed in section 2.5 we consequently selected a 2D mouse as input device. Besides of being robust and simple to setup (e.g., no need for calibration), other advantages are shorter pre-training phases and cost-effectiveness (Espingardeiro, 2012). The mouse holds three buttons, which the operator can use to trigger three high-level recovery behaviors (Figures 1, **9**) of the aerial telerobot (section 4.1).

## 4. System Implementation

To solve the challenging tasks that occur during aerial indoor exploration missions, we implemented the following components as part of our high-level teleoperation system:

- **Aerial telerobot** represented as a small-sized UAV. The UAV is equipped with a sensory setup to acquire RGB-video and depth data. The data is transferred to a desktop PC via the remote connection.
- **Human-robot interface** to facilitate control of the aerial telerobot by providing different views of the remote scene. Based on these views, the operator controls the telerobot in a supervisory manner via high-level interactions. It further holds the underlying system components that are responsible for flight control, 3D mapping, abstraction, detection of portals, and object detection in real-time. Remarkably, the components are vital for enabling the interactive scene topology of the human-robot interface. Thus, they are also essential to enable the high-level interactions (Explore!, Inspect!, Navigate!).
- **Input devices** that sends manual inputs to the human-robot interface. It is implemented as a simple and cost-effective mouse to interact with the RPG. To compare our system against traditional controls, we use a joypad controller for our user study (section 6.1).

**Figure 6:**
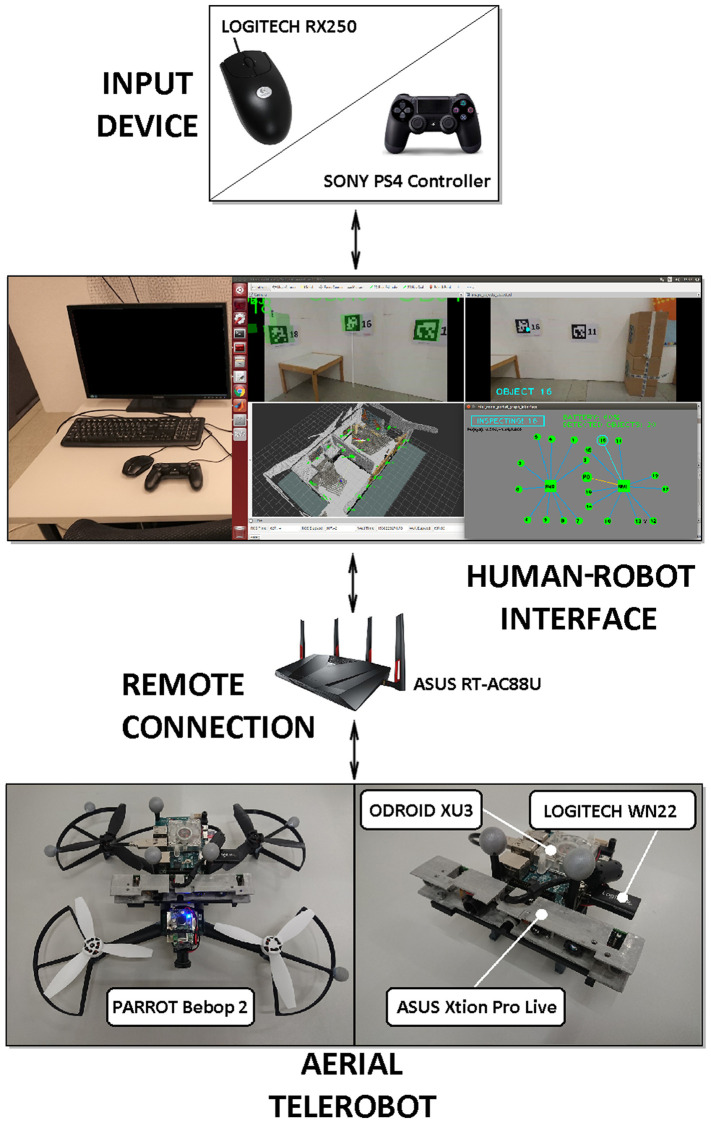
Implementation of the main components of the high-level teleoperation system: The UAV based on a Parrot Bebop 2 with onboard single-board-computer and sensory setup. The remote connection implemented as ASUS RT-AC88U wireless router. The GUI, including the RPG, implemented on a desktop PC in ROS. The input devices implemented as a Logitech RX250 mouse and a PS4 controller.

**Figure 7:**
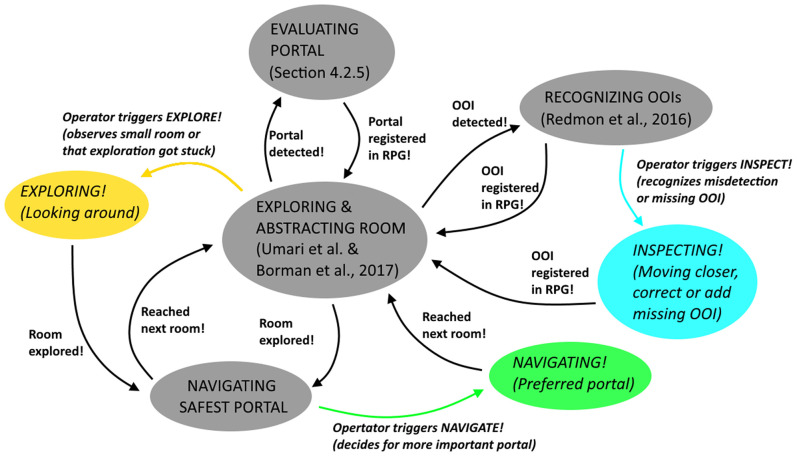
State diagram of the software framework of our teleoperation system.

While the physical setup of the teloperation system is shown in Figure 6, we give an overview of the software implementation (represented as state diagram), high-level interactions (Explore!, Inspect!, and Navigate!) and according recovery behaviours in Figure 7.

### 4.1. Aerial Telerobot (UAV)

For the aerial telerobot (UAV) of our system we use a modified Parrot Bebop 2 (Parrot, 2015). It is compact, suitable for narrow indoor spaces and offers open-source drivers (Monajjemi, 2015) for low-level flight control. For reliable experimentation, we attach retro-reflective markers for outside-in localization using an Optitrack Flex 13 infrared motion capturing system. An overview of the physical setup is shown in Figure 6.

With all on-board sensors attached, the outer dimensions of the UAV are 32.8 × 38.2 × 25 cm, and it weighs 700 g, with flight times of up to 10 min. On top of our UAV, we mount a customized RGBD sensor rig (250 g), consisting of an ASUS Xtion Pro Live sensor ($FOV_{hor.}=58^{∙}$, $FOV_{vert}=45^{∙}$) and a Logitech WN22 WiFi stick, connected via USB to an ODROID XU3 single-board computer. During our experiments, the UAV was navigating at a default flight height of *z*_*takeoff*_ = 1.25 m.

### 4.2. Human-Robot Interface and Input Devices

In this section, we give details about the human-robot interface which enables the operator to high-level control our teleoperation system. As one vital component it holds the RPG as interactive scene topology which is created based on the complex interplay of its underlying system components (section 4.3). While we motivate a supervisory control approach in section 3.2.1, in the following we discuss implementation details and the correspondence to the LOA. Furthermore, we give details about the baseline system with a joystick as input device in section 4.2.2. It runs on low automation level, and the operator has manual control over the system. We compare the two different systems against each other and report on results in section 6.1.

#### 4.2.1. High-Level Teleoperation (RPG Condition)

High-level teleoperation of our system is enabled by the RPG to let an operator effectively supervise our system on high LOA (Table 1). We intentionally do not provide low-level access, so that the operator is not burdened with demanding mission-critical tasks (ALFUS 1-6). This also means that the system must achieve all mission-critical tasks even in a challenging indoor environment. The system usually operates on highest LOA (ALFUS 10), but we let the operator switch to a lower collaborative level (ALFUS 7-9), if supervision is required. This is particularly relevant if the underlying system components do not perform satisfactorily, e.g., when object recognition fails (Materna et al., 2017).

**Figure 8:**
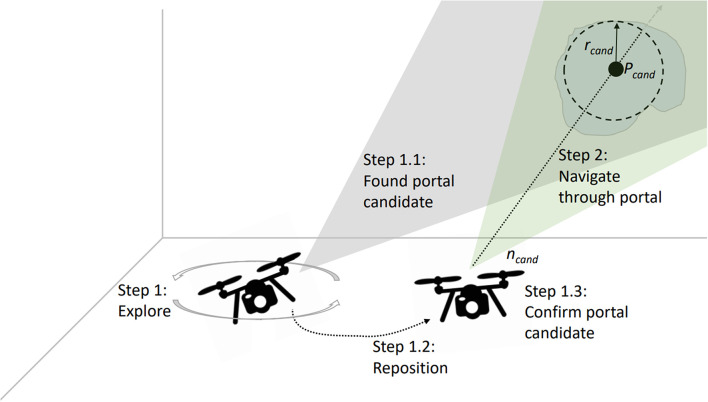
Method for detecting and evaluating potential safe portals directly from depth-image data. The UAV first explores the close environment and, if a safe-portal candidate is detected, positions itself to confirm that the portal candidate is safely traversable.

**Figure 9:**
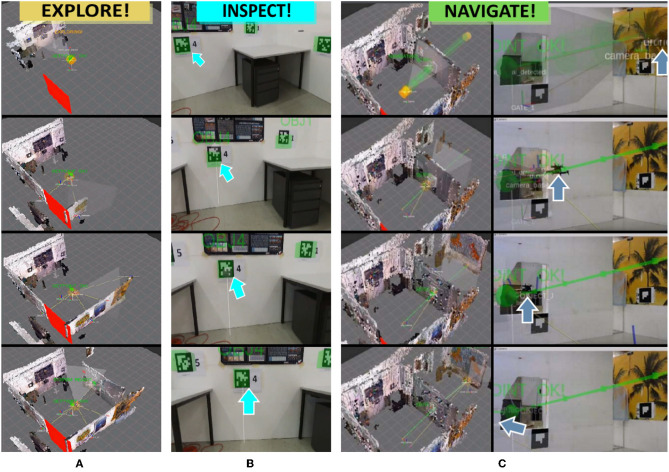
Exocentric virtual views of the aerial robotic system during execution of the recovery behaviors. **(A)** The UAV is commanded to explore its surrounding by flying a circular trajectory and simultaneously builds a 3D model of its environment from RGBD data. **(B)** The UAV is commanded to close inspect a detected object for verification. **(C)** The UAV is commanded to navigate through a safe portal to the adjacent room along an autonomously planned path, shown in green. The UAVs position is marked with blue arrows.

For the RPG (Figure 2), we combine a traditional egocentric view (on-board camera of the UAV) with an exocentric 3D map view. The views include visual aids for current pose of the UAV, view frustum of the onboard camera, the online reconstructed 3D environment and invalid flight zones. The purpose is provide a basic spatial understanding of the scene. We extend the ego- and exocentric views with an interactive topological view, the RPG. It consists of rooms (nodes) and portals (edges) to other rooms or OOIs (e.g., a fire extinguisher or a victim). OOIs registered in the RPG are highlighted in real-time. Once an interactive OOI is highlighted, the operator can use 2D mouse inputs to supervise the system via a reduced set of high-level interactions (**Explore!**, **Navigate!**, and **Inspect!**). This triggers recovery behaviors (Figures 8, 9) and implies switching from highest LOA (ALFUS 10) to collaborative level (ALFUS 7-9) (Figure 5).

The Explore! command lets the system more effectively uncover smaller rooms. During this task, our system autonomously detects OOIs and adds them as interactive nodes to the RPG topology. If a false detection occurs, the operator can use the Inspect! command to move closer. If one of the detected objects is selected, a safe path is generated between the current location of the UAV and the object. After the system navigates close to the false detection, the operator can inspect the situation in a close-up egocentric view and determine further action. During exploration of a room, also portals which are safe for navigation are detected and highlighted (section 4.3.4) automatically. Detected portals add a new node and edge to the RPG. In case of multiple detections, the operator is able to select a preferred portal to trigger navigation into the adjacent room (Navigate!). A picture sequence of the recovery behaviors is shown in Figure 9, whereas we present real-world flights in our Supplementary Video (section 7). Details about the physical setup of the aerial telerobot are discussed in section 4.1.

The goal of the RPG is to provide a topological map that is a human-like representation of the environment. Since it provides natural interactions for commanding the system and describing the environment, it facilitates and eases human-robot-interaction (Johnson, 2018). Moreover, its purpose is to reduce scene details in the presence of cluttered traditional views (left and middle Figure 3). However, the concept of the RPG has also limitations which we detail in section 5.

#### 4.2.2. Traditional Direct Teleoperation (JOY Condition)

To compare the effect of our high-level teleoperation system against a state-of-the-art baseline system, we implemented traditional joystick controls. For our study we define it as condition JOY. In this condition, the operator uses a joypad to command the UAV at lower ALFUS (Table 1) with a high-level of interaction on sides of the human operator (ALFUS 1-3). At this level, the system takes care of automatic take-off, position stabilization and landing. Besides, the operator is also responsible for mission-critical tasks.

To achieve fair comparison against the RPG, we added a visualization of flight zone boundaries to help the operator prevent collisions. The boundaries are displayed in the horizontal plane via a color-coded surface at the height of the UAV. Operators must not exceed this indicated boundary and get color-coded feedback, if they are close to exceed the maximum flight height. The surface turns orange, if the UAV is close to the height boundary, which means the distance of the geometric center of the UAV to the upper boundary is smaller than the height of the UAV. The surface turns red, if the distance is smaller than half of the height of the UAV, indicating that the operator has to steer the UAV downwards immediately.

The joypad is used in MODE-2 configuration, allowing the operator to give direct motion commands. In this configuration, the left rudder stick controls translational and rotational velocity of the z-axis of the UAV, and the right rudder stick gives acceleration inputs along the x-axis and y-axis of the UAV.

### 4.3. Underlying System Components

This sections describes the underlying components of our high-level teleoperation system. They are implemented as part of the human-robot interface on a Desktop PC and responsible for exploration, flight planning and navigation, 3D mapping of the environment, and highlighting of OOIs. Since they even enable the RPG as interactive scene topology, the effectiveness of our full system strongly depends on their performance. Thus, they must be emphasized as core for interaction. The aerial telerobot supports with automated indoor exploration and the human operator can trigger recovery behaviors via the RPG. Subsequently, if a non-mission-critical task fails, time performance could be improved. The recovery behaviors are designed in a supervisory manner so that the human operator can effectively supervise the system with difficult tasks on higher-level. Their purpose is illustrated throughout the following use cases:

- Explore: After take-off, the UAV autonomously starts exploring the current room using an RRT-based exploration method (Umari and Mukhopadhyay, 2017). If the operator decides that the room seems rather small or the exploration fails to fully explore the room, the operator can on demand trigger a simple recovery behavior. In that case the UAV explores the local environment by flying a circular trajectory. Once a room is fully explored we use the implementation of Bormann et al. (2016) for room-segmentation.
- Inspect: During exploration of a room, the telerobot autonomously detects portals and OOIs, like victims or fire-extinguisher. However, if the operator feels that an object was misdetected, the operator can command the telerobot to move closer to a detected OOI or portal for verification.
- Navigate: During room exploration, the telerobot detects portals which are safe to navigate. However, if multiple safe portals are detected, the human operator might intuitively prefer one portal over the other for navigation. In such cases the operator can manually trigger portal navigation.

#### 4.3.1. Room Exploration

At the beginning of every mission, the UAV ascends to a default flight height (section 6.1). After reaching the default height, the UAV starts to autonomously explore the local environment (Figure 8, Step 1). For local exploration of a single room, we use a frontier detection algorithm, based on rapidly-exploring random trees (Umari and Mukhopadhyay, 2017). If no failure cases occur, we consider the system to work on highest LOA (ALFUS 10).

Once the UAV takes off, we start detection of local frontiers by taking into account the occupancy map constructed online. First, we project 3D occupancy information into 2D, since this helps to clearly define boundaries of a single room. We project occupied cells into the 2D map. Second, we let a local frontier detector discover valid navigation points, which are derived from a rapidly growing random tree biased toward unexplored regions of the occupancy map. Third, we directly steer the UAV toward the detected point, incrementally exploring the local environment. These steps are repeated, until no new frontier points are detected and the room is locally fully explored. To abstract the local room and to further obtain room information we make use of the segmentation approach presented by Bormann et al. (2016). Note that we assume the range and FOV of our depth sensor to be wide enough to cover the close environment and detect potential obstacles, when navigating at default height. For simplicity, we assume that there are no additional obstacles between the UAV and the detected room boundaries. The operator is further able to manually override frontier detection by selecting the abstract room representation of the RPG (triggering Explore! and switching from highest- to collaborative LOA). This prompts the system to execute a more efficient circular trajectory.

#### 4.3.2. Room Navigation

To enable collision-free navigation through portals from one room to another, we use a global path planning approach based on probabilistic road maps (PRM) (Kavraki and Latombe, 1996). The global path planner generates a PRM based on the occupancy grid map (Hornung et al., 2013). The PRM is represented as a set of 3D points given in world coordinates. For an example of generated paths, please refer to Figure 9C.

The PRM is passed to a real-time motion planning framework (Gebhardt et al., 2016; Nägeli et al., 2017a,b). The motion planner involves a model predictive controller (MPC), which produces smooth motion trajectories for the UAV when moving along the global path (Supplementary Data Sheet 1). Following a receding-horizon MPC formulation, at each timestep Δ*t*, a locally optimal path with *N* steps and a duration of *NΔt* is computed. This optimization problem is re-evaluated at every sampling instance *T*_*s*_, leading to a closed-loop behavior. Thus, we make use of the disturbance rejection characteristics of the MPC to stabilize the UAV during the mission. Stabilization against turbulence is necessary when flying close to objects or passing through portals. The real-time motion planner is implemented in MATLAB (Robotics Toolbox), utilizing the FORCES Pro real-time solver (Domahidi and Jerez, 2013).

#### 4.3.3. Environmental Reconstruction

To provide the operator with basic environmental understanding during navigation (section 4.3.1), we make use of the RTABMap reconstruction framework (Labbe and Michaud, 2013, 2014). It represents the reconstructed geometry as a colored occupancy grid map and is capable of loop-closure detection. The grid map is created by fusing depth- and RGB-data from the onboard sensor setup of the UAV (Figure 6) and visualized in the exocentric view.

#### 4.3.4. Detecting and Highlighting Objects of Interest

For our experimental setup, we introduce different types of OOIs which are commonly present in exploration scenarios. These objects can be hazardous areas (location of fire extinguishers, broken power lines, gas leaks), human victims (embodied by human-like mannequins) or portals (potentially narrow passages), which connect adjacent rooms. The OOIs are automatically highlighted as virtual overlays in the GUI to direct the operators attention toward them. This requires automatic object detection and registration of the observed object positions in world coordinates. Noteworthy, we use a true relative scale between objects in the current design of the RPG. We detect objects either using the YOLO framework for object detection (Redmon et al., 2016) or by simply marking them with Apriltag markers (Olson, 2011) during the user study (section 6.1).

Indoor environments can typically be structured into wider open areas (rooms) and more narrow spaces (portals) connecting rooms (Kun et al., 2017). During the exploration task, our goal is to detect and visualize potential portals. Making rooms and portals explicit is vital in our scenario, since they support navigation. Interactive highlighting, helps operators to get a clearer understanding of the environment and make an educated decision on which part of the environment to explore next. The portal detection proceeds as follows (Figure 8): During exploration of the close environment (Step 1), we detect discontinuities in the depth images captured by the RGBD sensor. If the discontinuities form a closed, bounded region with minimum radius *r*_*cand*_ and depth *d*_*min*_ (measured from the centroid *P*_*cand*_ of the entry surface), the region is selected as a portal candidate (Step 1.1). This intermediate step is necessary to ensure the portal can be safely traversed, as looking at portals from larger offset angles would result in shadowed regions behind the portals. Based on the surface geometry of the portal candidate, we derive *P*_*cand*_ and the corresponding normal vector ${\vec{n}}_{cand}$. The normal ${\vec{n}}_{cand}$ is oriented perpendicularly to this entry surface and has its origin in *P*_*cand*_. In Step 1.2, the UAV is commanded to align the x-axis of its local coordinate frame *F*_*UAV*_ with ${\vec{n}}_{cand}$. The distance to the portal candidate *d*_*cand*_ is calculated based on the minimum radius *r*_*cand*_ and the narrower vertical field of view of the depth sensor *FOV*_*vert*_. *d*_*cand*_ can be expressed as *d*_*cand*_ = *r*_*cand*_/tan(*FOV*_*vert*_).

## 5. System Limitations

The teleoperation system presented in this work has also several limitations. The most important limitations are discussed in the following:

- Telerobot: Besides of there is room for improvement of our physical design (weight, size, and computational onboard power), also the ability to morph and adapt to challenging environments could be added. Speaking of passing narrow portals or gaps, highly dynamic maneuvers (Falanga et al., 2017) are currently not possible but could be interesting for future work. Another limitation of the telerobot is the exploration algorithm. While we make use of an RRT-based approach to explore a single room, but at constant flight height, a more powerful approach would involve full 3D exploration. Additionally, a gimbal could help to resolve constraints with the cameras limited FOV, making room exploration more efficient.
- Wireless Remote Connection: Due to the usage of an untethered remote connection between the telerobot and the human-robot interface, typical problems could occur like limited bandwidth and sudden connection dropouts. While in-field applications would require a much more sophisticated (and expensive) setup, in our implementation we considered commodity hardware only. However, it must be stated that due to usage of a powerful WiFi router, comparably short ranges, and non-overlapping/non-populated channels the effects during the user study could be reduced to a minimum.
- Supervisory Control of high-level tasks: The supervisory control approach of our system aims for effectively resolving failures of high-level tasks. However, this is only valid if the telerobot is capable of handling all low-level mission-critical tasks without failure.
- Human-Robot Interface: An essential component of our human-robot interface is the RPG, serving as interactive scene topology. The focus of its design is to supplement traditional views by supporting affordances and reducing scene detail. Thus, overwhelming the operator should be avoided. However, several aspects could not be considered in this work. While in our current RPG design we use a true relative scale of rooms, portals and objects, we did not elaborate on different layouts of the objects inside the RPG view or adapting its orientation relative to the 3D view. We also did not yet investigate on proper placement of the simplistic 2D objects in case they overlap or on altering their shapes and size. Future work would also include a zooming function for wider areas and adding important details on demand. Such helper functions could display size and volume of the selected room or distance between the telerobot and according OOI if selected with the input device.
- Input device: The design of our teleoperation system supports a robust and simple-to-use input device which is also cost effective. As a consequence we utilize a traditional 2D mouse with three buttons. These are dedicated to our three high-level interactions (Figure 1) to trigger recovery behaviors. However, the design of interactions and button mappings could be still improved by evaluating different layouts toward optimum usability. Further, utilizing a mouse with more degrees of freedom (Razor, 2015) could improve support for multi-floor exploration or manual steering of a camera gimbal with the attached joystick.
- Multi-floor environments: To be able to explore multi-floor environments, our system would require further components. For instance, the system would need to be able to detect stairways (Delmerico et al., 2013). In addition, the robustness of the untethered remote connection would have to be improved. While the implementation of our current system uses commodity hardware, systems with increased power and higher penetration of structures are for example presented by Patra et al. (2007). Additionally, like introduced for nuclear power plant inspection (Nagatani et al., 2013; Zhang et al., 2018), one or multiple additional telerobots could be used as mobile wireless transmission relays, retaining reliable communication.

## 6. User Study

**Figure 10:**
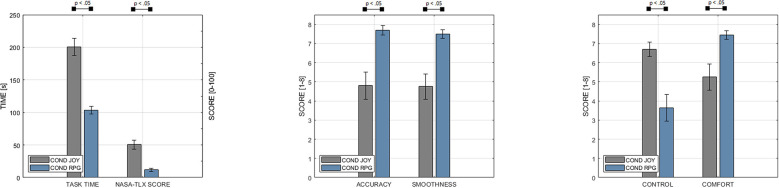
Our study results indicate significantly decreasing task times (Scale: 0–250 s) and decreasing NASA-TLX score (Scale: 0–100) with our high-level teleoperation system (condition RPG). Based on an even 8-point Likert scale (Agreement-Scale: 1 Strongly Disagree - 8 Strongly Agree), we managed to retain general comfort during operation, compared to our baseline system with traditional joystick controls (condition JOY). In addition, participants reported increasing accuracy of control and smoothness of control.

The purpose of our study is to investigate the effect of our high-level teleoperation system on operator performance during real-world exploration missions. We considered the different teleoperation systems as strongest baseline for our study conditions, whereas we compare our high-level teleoperation system, including the RPG (section 4.2.1), against a traditional baseline system with direct controls (section 4.2.2). Table 2 gives an overview of the experimental conditions, type of systems, and view modes, whereas (Figure 10) summarizes results of our user study.

**Table 2:** User study conditions.

	**JOY condition**	**RPG condition**
**Type of control**	**Traditional direct**	**High-level supervisory**
LOA	1–3	7–10
RPG view	No	Yes
EXO view	Yes	Yes
EGO view	Yes	Yes

A core aspect of our study is that, despite a variety of related work has shown semi-autonomous systems positively effecting task performance, however it is unclear if this holds in a realistic setting where a system has to generate an interactive abstract topological scene view in real-time during flight missions. While operators with traditional direct controls can issue commands based on their quasi-instantaneous human cognition, operators of the semi-autonomous system need to wait until it processes, abstracts and outputs (visualizes) the abstracted information. This raises the question if such systems are still able to improve task performance over traditional control approaches in a realistic setting, where operators potentially need to wait until information is available in the topological view.

### 6.1. Experimental Design

In the following sections, we summarize the experimental design of our user study, including study conditions and tasks. Besides, we give details about study procedure, participants and accordance of the study to the local legislation.

#### 6.1.1. Conditions

The main objective of our study was to assess the effect of the two user interface conditions, RPG and JOY, on operators task times, mental load and general comfort during a real-world indoor exploration mission. We based our study on **within-subjects design** and varied the order of the conditions using **full counterbalancing**. We defined task completion time, mental load and general comfort of the operator as main task performance metrics. We formulated the following hypothesis for the user study and report on results in section 6.2:

- H1: The operator's task time decreases in RPG.
- H2: The operator's mental load decreases in RPG.
- H3: The operator's general comfort increases in RPG.

#### 6.1.2. Tasks

**Figure 11:**
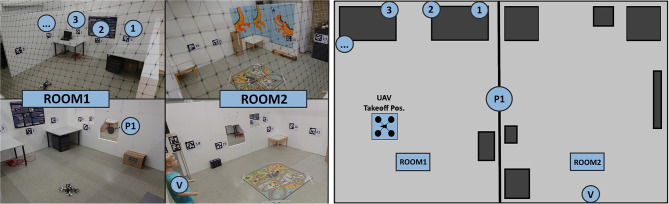
(**Left**) Physical setup for our experimental evaluation. Note the two rooms, connected via a safe portal and the objects of interest including a victim. (**Right**) The same environment, represented as a floor-plan in 2D.

According to Bhandari et al. (2015), typical indoor exploration tasks involve investigation of the unknown environment and evaluation of hazardous areas to minimize human risk. We designed our study so that participants had to fulfill similar tasks in our experimental setup (Figure 11). We assumed a situation where the operator is far from the indoor space and has no prior knowledge of it. To ensure a basic degree of validity, we discussed the design of our experimental task-design with a local fire brigade. As a conclusion, the firefighters confirmed the validity of our task design and further emphasized usefulness of our system for assessment of a stable but still potentially dangerous situation. As an example use case, they specified the on-site inspection of a damaged chemical recovery boiler where an imminent explosion cannot be ruled out.

The indoor exploration task of our study comprises three subtasks, which had to be completed by each participant in each of the conditions. During this task, participants had to fully explore the environment and find all OOIs. In particular, participants were told to:

- Find all 19 hazardous areas marked with fiducial markers.
- Find the safe portal.
- Find the victim.

The placement of objects was altered in a controlled fashion to avoid learning effects. An overview of the experimental indoor environment is given in Figure 11.

#### 6.1.3. Procedure

Before each experimental session, an introduction to the teleoperation system was given to the participant by the experimenter. Preliminary questions were asked to identify eye-sight restrictions. The evaluation procedure of each experimental condition can be split into three phases. In a training phase, participants learned to use the system of the specific condition. This phase ended when participants reported to be comfortable in using the system. In the second phase, participants had to accomplish the indoor exploration task as fast as possible. For each participant, we captured screen recordings and measured the task completion time using a stop watch. The task was considered to be completed when the system detected all safe portals, hazardous areas and victims (RPG condition) or users verbally confirmed to the experimenter that they found all of those objects (JOY condition). In both conditions, users were aware of the number of objects they already identified as well as of the objects they still need to find. Finally, participants were asked to fill out a NASA-TLX (Hart and Staveland, 1988) task-load questionnaire (Scale: 0–100) as well as a custom questionnaire with respect to their experience in the respective condition. The custom questionnaire contained 8-point Likert items (ranging from 1, “strongly disagree," to 8, “strongly agree") asking participants about accuracy and smoothness of control as well as their perception of control over the system and their general comfort during the task.

#### 6.1.4. Participants

A total of 23 participants were invited, 20 of them successfully finished the given tasks in all conditions. Three participants had to stop the study due to technical problems and their results have been excluded. We invited 17 male participants and 3 female participants which were either students or researchers in the field of computer science or electrical engineering at Graz University of Technology (age: *M* = 27.6, *SD* = 3.98).

#### 6.1.5. Ethics Statement

The presented study was carried out in accordance with the World Medical Association's Declaration of Helsinki, as revised in 2013 (HELSINKI, 2013). The study did not involve any medical experiments and further, no biometric data was taken from participants. We did not take any personal data from participants besides age, whereas all taken data was fully anonymized. In general, the study was conducted in accordance with the local General Data Protection Regulation (GDPR) in Austria and all participants gave written informed consent via an IRB consent-form (Supplementary Table 1). As per the local legislation, no IRB approval was required for our particular study type.

### 6.2. Results

In each of our 20 sessions, we tested the teleoperation system in both conditions, JOY and RPG. This resulted in a total of 40 valid runs. For each participant, we took screen recordings and measured the task completion time during the flight. After finishing the flight for one condition, participants were asked to fill out the NASA-TLX score as well as a custom questionnaire (Supplementary Data Sheet 2). This questionnaire contained several 8-point Likert items asking participants about the accuracy of control, the smoothness of control, their perception of control over the system and their comfort in general during the task. We report mean, standard deviation and interval estimates for a 95% confidence interval (Supplementary Table 2). For significance testing, we use a paired samples t-test for task execution time as the data is normally distributed. All other measures are compared using Wilcoxon signed-rank test as questionnaire responses are non-parametric.

The main findings of our study are summarized in Figure 10. Statistical testing revealed that the task completion time was significantly lower for the RPG- (*M* = 103.7*s, SD* = 13.7*s*) compared to JOY [*M* = 200.1*s, SD* = 30.58, *t*_(19)_ = 12.01, *p* < 0.001]. In addition, a significant effect of conditions on mental load, as determined by NASA-TLX, has been revealed (*Z* = 210.0, *p* < 0.001). Again, RPG (*M* = 11.75, *SD* = 6.43) caused a significantly lower mental load than JOY (*M* = 50.71, *SD* = 16.41).

In our custom questionnaire, we asked participants about their perception of the tested user interface. Unsurprisingly, the perceived level of control in conditions decreased with the increasing LOA from JOY (*M* = 6.7, *SD* = 0.87) to RPG (*M* = 3.65, *SD* = 1.63). Wilcoxon signed-rank test showed that these differences are significant (*Z* = 169.0, *p* < 0.001). In contrast, participants perceived RPG (*M* = 7.7, *SD* = 0.57) to be significantly more accurate than JOY (*M* = 4.8, *SD* = 1.67, *Z* = 0.0, *p* < 0.001). Similarly, perceived smoothness of control was higher for RPG (*M* = 7.5, *SD* = 0.51) compared to JOY (*M* = 4.75, *SD* = 1.55). Again, differences are significant (*Z* = 0.0, *p* < 0.001). Finally, perceived general comfort was significantly higher in the RPG condition (*M* = 7.45, *SD* = 0.51), compared to JOY (*M* = 5.25, *SD* = 1.62), with (*Z* = 0.0, *p* < 0.001). This lets us accept H3, which is supported by a significantly higher task completion confidence in RPG (*M* = 7.8, *SD* = 0.41), compared to JOY (*M* = 6.8, *SD* = 1.06, *Z* = 0.0, *p* < 0.001).

### 6.3. Discussion

Overall, we were able to support all of our three hypotheses, implying that our high-level teleoperation system is successful in supporting the operator during aerial exploration missions in challenging indoor environments. Remarkably, our teleoperation system reduced task execution times by 48.28% and task load by 76.82% compared to the JOY condition. Moreover, results indicate an increase in general comfort by 41.90%. We attribute the significant differences between conditions to the interplay of the RPG-view and the autonomous system. However, further research is necessary to differentiate the influence of the autonomous system and the topological scene view on results.

Although, participants conducted real-world flights to solve the posed exploration task, the study took place in a controlled environment. For instance, localization of the UAV was achieved with a motion capture system. However, on-board localization methods like SLAM have proven to be sufficiently accurate and fast to be used for UAV position estimation (Weiss et al., 2011; Mur-Artal and Tardós, 2017). In addition, due to limited lab space, the environment of our study did only comprise two rooms. Nonetheless, we believe that differences between conditions further evolve in favor of our system in wider- or multi-floor environments. The reason is that it is evidently harder to gain a good spatial understanding of larger compared to smaller environments. Thus, operators will benefit more from the RPG view in larger spaces, as the RPG abstracts the environment in an easy-to-understand manner. Furthermore, the task of our study was a simplification of complex real-world search and rescue missions. However, it is likely that our system even better supports operators in more complex task scenarios. For instance, research has shown that topological views, like the RPG, are beneficial if an environment is fully explored and operators are required to repetitively navigate between OOIs (Johnson, 2018). With regards to our system, the reinspection of an OOI could easily be performed by triggering its visualization in the RPG. The telerobot would then autonomously renavigate to the specific room and object. Due to mentioned reasons, we argue that, despite limitations, our experimental setting is an ecologically valid approximation of a real-world exploration mission.

Summarizing, our study has shown that high-level teleoperation systems with an on-the-fly created interactive scene topology are still able to better support operators in real-world settings, compared to systems using traditional controls.

## 7. Conclusion and Outlook

In our work, we demonstrate a fully working teleoperation system for aerial exploration missions. It improves task performance by using an interactive scene topology, whereas related work motivates using topological representations for robotic teleoperation. However, in contrast to related work, we for the first time investigate on how task performance is effected if the topology is created in real-time during actual indoor flight missions. The overall goal of our system was to reduce task times and mental load of the operator while conserving general comfort. To elaborate on the expected improvement, we evaluated our teleoperation system with a user study under real-world conditions. We compared our high-level teleoperation system against a traditional baseline system with joystick control. Results indicate that our system positively effects task performance and operators comfort during aerial exploration of challenging indoor environments.

In future work we would like to address the limitations of our system (section 5) and conducted user study (section 6.3). Further we would like to evaluate our system in larger or even multi-floor environments, for which abstraction has a potentially larger benefit in terms of overall task performance.

## Data Availability Statement

All datasets generated for this study are included in the manuscript/Supplementary Files.

## Ethics Statement

The presented study was carried out in accordance with the World Medical Association's Declaration of Helsinki, as revised in 2013. The study did not involve any medical experiments and further, no biometric data was taken from participants. We did not take any personal data from participants besides age, whereas all taken data was fully anonymized. In general, the study was conducted in accordance with the local General Data Protection Regulation (GDPR) in Austria and all participants gave written informed consent via an IRB consent-form. As per the local legislation, no IRB approval was required for our particular study type.

## Author Contributions

WI, CG, OH, and DS contributed conception of the user interface and design of the study. WI implemented the user interface and wrote the first draft of the manuscript. WI and TN designed and implemented the aerial robotic system. WI and CG performed the statistical analysis of the study. WI, CG, FF, and DS wrote sections of the manuscript. All authors contributed to manuscript revision, read, and approved the submitted version.

### Conflict of Interest

The authors declare that the research was conducted in the absence of any commercial or financial relationships that could be construed as a potential conflict of interest.

## Footnotes

^1^The reported levels of automation (LOA) are based on the ALFUS framework of Huang et al. (2005a,b).

## Supplementary Material

An extended version of the Supplemental Video 1 can be found via https://youtu.be/1vXYoes1-lY.

Supplementary Data Sheet 1 Realtime motion planning algorithm.

Supplementary Data Sheet 2 User study custom questionnaire.

Supplementary Table 1 User study IRB consent form.

Supplementary Table 2 User study raw data.
