# Supplementary material for: High-Level Teleoperation System for Aerial Exploration of Indoor Environments
Source: Front Robot AI. 2019 Oct 23;6:95. doi: 10.3389/frobt.2019.00095 (PMC7805862; doi:10.3389/frobt.2019.00095)
Supplement: Supplementary Data Sheet 1 — Realtime motion planning algorithm. [file Data_Sheet_1.pdf]

Supplementary Material - Real Time Motion Planning

1 ABSTRACT

For completeness and reproduceability, this document provides details about the motion planning algorithm of our high-level teleoperation system. The underlying method is called Model Predictive Contouring Control (MPCC) and commonly used for applications like control of robotic arms (Faulwasser et al., 2017), but also for autonomous driving (Rosolia et al., 2017). It is used for collission-free room-to-room navigation of the systems aerial robot triggered by the high-level interactions of the teleoperator in the room-portal graph (RPG).

2 NOTATION

We provide a summary of notations related to the motion planning methods used for the presented teleoperation system. They are shown in Tbl. S1. The notations and descriptions of the mathematical relations are based on the work of Nägeli et al. (2017a,b).

Table S1. Summary of notations used in this paper.

Symbol	Description
$\mathbf{p_q}, \dot{\mathbf{p}}_\mathbf{q}$	Quad position and velocity
v_z	Quad velocity in z-direction
ω_{q_z}	Quad angular velocity around body-z
$\mathbf{p_{q_i}}$	Position of quad i
Φ_q, Θ_q, Ψ_q	Quad roll, pitch, yaw
ϕ_q, θ_q	Quad desired roll, pitch
$R_{\psi_q}(\psi_q)$	Quad yaw matrix
$\mathbf{x}, \mathbf{u}$	Quad states and inputs
T	Planned trajectory
**N**	Prediction horizon in MPCC problem
T_s	Sampling time

3 QUADROTOR DYNAMICS MODEL

The state of the quadrotor is given by its position $p_q \in \mathbb{R}^3$, its velocity $\dot{p}_q \in \mathbb{R}^2$ and its orientation, i.e. roll, pitch and yaw $(\Phi_q, \Theta_q, \Psi_q)$. We define the state of the system, represented by the quadrotor, which can be denoted as

$$\mathbf{x} = \left[p_q, \dot{p}_q, \Phi_q, \Theta_q, \Psi_q\right] \in \mathbb{R}^\mathbf{8} \tag{S1}$$

Based upon the control inputs which are available with the Parrot Bebop 2 SDK, the control inputs to the system can be defined with the vector

$$\mathbf{u} = \left[v_z, \phi_q, \theta_q, \omega_{\psi_q}\right] \mathrm{R}^\mathbf{4} \tag{S2}$$

where v_z is the velocity of the quadrotor in the body-z axis, ϕ_q, θ_q are the desired roll and pitch angles of the quadrotor, respectively, ω_{ψ_q} is the angular speed around the body-z axis. The horizontal velocities are not directly controlled. A first order low-pass Euler approximation is used to describe the dynamics of the

quadrotor. The translational dynamics are then given by

$$\dot{\mathbf{p}}_{\mathbf{q}} = \left[\dot{p}_{q_{x,y}}, v_z\right] \tag{S3}$$

$$\ddot{\mathbf{p}}_{\mathbf{q}} = \left[\ddot{p}_{q_{x,y}}, 0\right] \tag{S4}$$

$$\ddot{\mathbf{p}}_{\mathbf{q_{x,y}}} = R_{\Psi_q}(\Psi_q) \begin{bmatrix} -\tan\Psi_q \\ \tan\Phi_q \end{bmatrix} g - C\dot{\mathbf{p}}_{q_{x,y}} \tag{S5}$$

where $g = 9.81\frac{m}{s^2}$ is the earths gravity, $R_{\psi_q}(\psi_q) \in SO(2)$ is the rotation matrix containing the yaw rotation of the quadrotor only and C is the drag coefficient at low speeds. The rotational dynamics of the quadrotor are then given with

$$\dot{\Phi}_q = \tau_a(\phi_q - \Phi_q) \tag{S6}$$

$$\dot{\Theta}_q = \tau_a(\psi_q - \Psi_q) \tag{S7}$$

$$\dot{\Psi}_q = \omega_{\psi_q} \tag{S8}$$

4 DISTURBANCE REJECTION (MPCC)

Smooth path approximation is done by minimizing projection of the drones position p_q onto the path:

$$\theta^* = \arg\min_{\theta} \|\mathbf{s}(\theta) - \mathbf{p}_q\| \tag{S9}$$

The projection represents the closest distance to the generated path, which is usually denoted as contouring error $\in^C$. Note that the contouring error is not suitable for formulation in the local optimization problem, because it needs to be optimized by itself along the entire global path. To this purpose it is necessary to approximate $\in^C$ by separating it into contouring and lag error. The lag error $\in^l = \int_{\theta^\star}^{\theta_k} s(x)dx$ is an integral over the path segment between the desired location on the path $\theta^\star$ and the location θ, found by solving the MPCC problem.

4.1 Error measurements

Our approach is also transferable to the 3D case, involving separation of lag and contour error. The errors $\in^l$ and $\in^c$ are approximated by projecting the current position $\mathbf{p_q}$ onto the tangent vector $\mathbf{n}$, with origin at the current path position $\mathrm{s}(\theta)$. The relative vector between $\mathbf{p}_q$ and the tangent point s can be defined as $\mathbf{r}_{p_q^s} := \mathrm{s}(\theta) - \mathbf{p}_q$. Further the derivative of the path $\mathrm{s}(\theta)$ with respect to the path parameter θ is defined as: $\mathbf{s}' := \frac{\partial \mathrm{s}(\theta)}{\partial\theta}$ which defines the normalized tangent vector $\mathbf{n} = \frac{\mathbf{s}'}{\|\mathbf{s}'\|}$. The approximation of the lag error is then given with:

$$\hat{\in}^l(\mathbf{p}_q, \theta) = \left\|\mathbf{r}_{\mathbf{p}_q S}^T \mathbf{n}\right\| \tag{S10}$$

Approximation of the contour error is given by:

$$\hat{\in}^c(\mathbf{p}_q, \theta) = \left\|\mathbf{r}_{\mathbf{p}_q\mathbf{S}} - (\mathbf{r}_{\mathbf{p}_q\mathbf{S}}^T \mathbf{n})\mathbf{n}\right\| \tag{S11}$$

After definition of the errors, we can define a cost function $c_p : \mathrm{R}^4 \lim \mathrm{R}_+$ which represents the trade-off between path-following accuracy and progress $\dot{\theta}$ along the path:

$$c_p(\mathbf{p}_q, \theta, \dot{\theta}) = \begin{bmatrix} \hat{\in}^l(\mathbf{p}_q, \theta) \\ \hat{\in}^c(\mathbf{p}_q, \theta) \end{bmatrix}^T \mathbf{Q} \begin{bmatrix} \hat{\in}^l(\mathbf{p}_q, \theta) \\ \hat{\in}^c(\mathbf{p}_q, \theta) \end{bmatrix} - \beta\dot{\theta} \tag{S12}$$

where $\mathbf{Q} \in \mathrm{S}^2_+$ is a (typically diagonal) positive definite weight matrix, chosen by the user, and $\beta \geq 0$ is a scalar weight, which, in our case, is chosen to be 0. If $\beta = 0$, the UAV is forced to stay on the virtual rail, and its position along the path is free running.

If $\hat{\in}^l(\mathbf{p}_q, \theta)$ becomes small, the approximation quality of the contour error increases ($\hat{\in}^l(\mathbf{p}_q, \theta) \lim 0$, then $\hat{\in}^c(\mathbf{p}_q, \theta) \approx \in^c$). Therefore, a high penalty is chosen for $\hat{\in}^l(\mathbf{p}_q, \theta)$. For our purpose this is beneficial since we want the UAV to fly through narrow passages safely.

4.2 MPCC Problem

In the following, we discuss the MPCC problem more in detail. The solution of this N-step finite horizon constrained non-linear optimization problem are the trajectory and control inputs of the UAV at each time step t. The problem can be denoted as

$$\min_{u,x,\theta,\theta^\star} \sum_{k=0}^{N-1} (J_k + \mathbf{u_k}^T \mathbf{R} \mathbf{u_k}) + a_N J_N \tag{S13}$$

whereas and a stage cost J_k, including cost terms as performance indices for path generation and following.

$\mathbf{x_0} = \hat{\mathbf{x}}(\mathbf{t})$	Initial state
$\theta_0 = \hat{\theta}(t)$	Initial path parameter
$\mathbf{x}_{k+1} = f(\mathbf{x_k}, \mathbf{u_k})$	Dynamics
$\theta_{k+1} = \theta_k + \dot{\theta}_k T_s$	Progress on virtual rail
$0 \leq \theta_k \leq L$	Virtual rail length
$\mathbf{x}_k \in \mathrm{X}$	State constraints
$\mathbf{u}_k \in \mathrm{U}$	Control input constraints

The stage cost, in our case, can be expressed as

$$J_k = a_p c_p(\mathbf{p}_{q_k}, \theta_k, \dot{\theta}_k) \tag{S14}$$

where $\mathbf{R} \in \mathbb{S}_+^{\mathbf{n_u}}$ is a positive definite penalty matrix, avoiding excessive use of the control inputs. The vector $\hat{x}(t)$ and the scalar $\hat{\theta}(t)$ represent the measured or estimated values of the current states $\mathbf{x}$ and θ, respectively. The scalar T_s is the sampling interval. The scalar $a_N > 0$ is a weight parameter used to weight the terminal cost J_N on the final stage. This is a common approach in finite horizon schemes, such as MPCC, as a replacement for long horizons, approximating the infinite horizon solution. The UAV is actuated using optimal inputs from the first iteration $\mathbf{u_0}$. A new trajectory is recomputed at each time step t, taking updated sensor data and rail configuration into account.

REFERENCES

Faulwasser, T., Weber, T., Zometa, P., and Findeisen, R. (2017). Implementation of nonlinear model predictive path-following control for an industrial robot. *IEEE Transactions on Control Systems Technology* 25, 1505–1511

Nägeli, T., Alonso-Mora, J., Domahidi, A., Rus, D., and Hilliges, O. (2017a). Real-time motion planning for aerial videography with dynamic obstacle avoidance and viewpoint optimization. *IEEE Robotics and Automation Letters* 2, 1696–1703. doi:10.1109/LRA.2017.2665693

Nägeli, T., Meier, L., Domahidi, A., Alonso-Mora, J., and Hilliges, O. (2017b). Real-time planning for automated multi-view drone cinematography. *ACM Trans. Graph.* 36, 132:1–132:10. doi:10.1145/3072959.3073712

Rosolia, U., Carvalho, A., and Borrelli, F. (2017). Autonomous racing using learning model predictive control. In *2017 American Control Conference (ACC)*. 5115–5120. doi:10.23919/ACC.2017.7963748
