# Supplementary material for: High-Level Teleoperation System for Aerial Exploration of Indoor Environments
Source: Front Robot AI. 2019 Oct 23;6:95. doi: 10.3389/frobt.2019.00095 (PMC7805862; doi:10.3389/frobt.2019.00095)
Supplement: Supplementary Data Sheet 2 — User study custom questionnaire. [file Data_Sheet_2.PDF]

USER STUDY - QUESTIONNAIRE

Questionnaire is given to participant AFTER EACH CONDITION!

* Required

1. **Condition:** *

   Mark only one oval.

   ( ) 1

   ( ) 2

2. **Please fill in first letters of your name or an ID:** *

   ____

3. **What was your NASA TLX Score?** *

   ____

4. **What was your time performance in SECONDS?** *

   ____

5. **I felt confident that I fully completed the given task.** *

   Mark only one oval per row.

	1 (Strongly Disagree)	2 (Disagree)	3 (Moderately Disagree)	4 (Mildly Disagree)	5 (Mildly Agree)	6 (Moderately Agree)	7 (Agree)	8 (Strongly Agree)
Scale	( )	( )	( )	( )	( )	( )	( )	( )

6. **I felt that I was in control of the system.** *

   Mark only one oval per row.

	1 (Strongly Disagree)	2 (Disagree)	3 (Moderately Disagree)	4 (Mildly Disagree)	5 (Mildly Agree)	6 (Moderately Agree)	7 (Agree)	8 (Strongly Agree)
Scale	( )	( )	( )	( )	( )	( )	( )	( )

7. **I felt comfortable with this type of control during the given task.** *

   Mark only one oval per row.

	1 (Strongly Disagree)	2 (Disagree)	3 (Moderately Disagree)	4 (Mildly Disagree)	5 (Mildly Agree)	6 (Moderately Agree)	7 (Agree)	8 (Strongly Agree)
Scale	( )	( )	( )	( )	( )	( )	( )	( )

8. **I felt comfortable in general during the given task.** *

Mark only one oval per row.

	1 (Strongly Disagree)	2 (Disagree)	3 (Moderately Disagree)	4 (Mildly Disagree)	5 (Mildly Agree)	6 (Moderately Agree)	7 (Agree)	8 (Strongly Agree)
Scale	◯	◯	◯	◯	◯	◯	◯	◯

9. **I felt that the control of the system during the given task was accurate.** *

Mark only one oval per row.

	1 (Strongly Disagree)	2 (Disagree)	3 (Moderately Disagree)	4 (Mildly Disagree)	5 (Mildly Agree)	6 (Moderately Agree)	7 (Agree)	8 (Strongly Agree)
Scale	◯	◯	◯	◯	◯	◯	◯	◯

10. **I felt that the control of the system during the given task was smooth.** *

Mark only one oval per row.

	1 (Strongly Disagree)	2 (Disagree)	3 (Moderately Disagree)	4 (Mildly Disagree)	5 (Mildly Agree)	6 (Moderately Agree)	7 (Agree)	8 (Strongly Agree)
Scale	◯	◯	◯	◯	◯	◯	◯	◯

11. **I felt that the control of the system was mentally demanding during the given task .** *

Mark only one oval per row.

	1 (Strongly Disagree)	2 (Disagree)	3 (Moderately Disagree)	4 (Mildly Disagree)	5 (Mildly Agree)	6 (Moderately Agree)	7 (Agree)	8 (Strongly Agree)
Scale	◯	◯	◯	◯	◯	◯	◯	◯

12. **I felt that the ROOM-PORTAL-GRAPH was helpful for completing the given task (Only valid for CONDITION 2!).** *

Mark only one oval.

◯ Yes

◯ No

Powered by
Google Forms
