# Supplementary material for: High-Level Teleoperation System for Aerial Exploration of Indoor Environments
Source: Front Robot AI. 2019 Oct 23;6:95. doi: 10.3389/frobt.2019.00095 (PMC7805862; doi:10.3389/frobt.2019.00095)
Supplement: Supplementary Table 1 — User study IRB consent form. [file Table_1.DOCX]

# User Study-Consent Form For Prototype Evaluation Of A

“High-Level Teleoperation System For Aerial Exploration Of Indoor Environments” (Page 1 of 2)

This Consent form is based on the IRB sample consent form, also recommend by the Cornell University:

<https://www.irb.cornell.edu/forms/sample.htm>

You are being asked to take part in a research study to test the performance of a novel teleoperation system. The prototype system (High-Level Teleoperation System) was developed at the Institute of Computer Graphics and Vision (ICG) at the Graz University of Technology. We measure task-efficiency of the system during and your mental load after the study which reflects overall performance of the prototype. In addition you have to fill out a questionnaire which helps to substantiate performance evaluation. We clearly state that we measure performance of the prototype system but not your performance. We are asking you to take part because you voluntarily agreed to participate in this study. Please read this form carefully and ask any questions you may have before agreeing to take part in the study.

**What the study is about:** The prototype consists of a teleoperated aerial robot (small-sized flying drone) which can be commanded with two different types of user interfaces. Via the user interfaces you will be able to steer the drone in two study conditions. This is either with a Joypad or via Point-and-Click Mouse-Inputs. The purpose of this study is to measure how the prototype system can increase task efficiency during an indoor exploration mission, whereas you have to solve typical task like navigating the small drone inside a narrow indoor environment, finding objects of interest and identifying artificial victims. You don’t need any prior experience with operation of small-sized drones and you don’t need to be experienced with teleoperation to take part in this study. However we require that you don’t have any sight-restrictions (color-blindness, etc.) and that you are not visually impaired in general.

**What we will ask you to do:** If you agree to be in this study, we will ask you to teleoperate the small sized drone in two different user-study conditions which involve two different types of user-interfaces. You have to command the drone and fully explore an indoor environment, whereas you have to solve 3 task categories:

- Find all 19 hazardous areas marked with fiducial markers (Apriltags)
- Find the safe portal
- Find the artificial victim (marked with a human puppet)

After each study condition we will ask questions about your mental load during the study (Nasa-TLX Test), perceived level of control, accuracy and smoothness of control and comfort during teleoperation. With your permission, we would also like to take your age and anonymized screen-recordings of the tasks to measure your task-time. The study will take about 45 minutes to complete.

**User Study-Consent Form For Prototype Evaluation Of A**

“High-Level Teleoperation System For Aerial Exploration Of Indoor Environments” (Page 2 of 2)

**Risks and benefits:** We do not anticipate any risks to you participating in this study other than those encountered in day-to-day life. There are no benefits to you. The study is very helpful to us to assess the current performance of our prototype and improve it in the future.

**Compensation:** You do not get any compensation for participating in the study. Participation is completely voluntary.

**Privacy:** All data that we take from you during the study will be fully anonymized. All your answers will be confidential. The records of this study will be kept private. In any sort of report we make public we will not include any information that will make it possible to identify you. Research records will be kept secure; only the researchers will have access to the records. If we screen-record your study-participation, everything is anonymized and we will destroy it after task-times have been recorded. In general, all personal data is treated according to the local General Data Protection Regulation (GDPR) in Austria (DSGVO).

**Taking part is voluntary:** Taking part in this study is completely voluntary. You may skip any questions that you do not want to answer. If you decide not to take part or to skip some of the questions, it will not affect your current or future relationship with Graz University of Technology. If you decide to take part, you are free to withdraw at any time.

**If you have questions:** The researchers conducting this study are W. A. Isop. Please ask any questions you have now. If you have questions later, you may contact W. A. Isop at isop@icg.tugraz.at. You can reach W. A. Isop at Inffeldgasse 16/II Room E.3.27. If you have any questions or concerns regarding your rights as a subject in this study, you may contact the local ethics commission in Graz (Austria) via (+43/0)316/385-13928 or access their website at <https://www.medunigraz.at/ethikkommission/Graz/> where you could also report any concerns or complaints anonymously.

You will be given a copy of this form to keep for your records.

**Statement of Consent:** I have read the above information, and have received answers to any questions I asked. I consent to take part in the study.

Your Signature ___________________________________ Date **XX.09.2018**

Your ID ______________ Your Age _______________

In addition to agreeing to participate, I also consent to having my participation screen-recorded.

Your Signature ___________________________________ Date **XX.09.2018**

Signature of person obtaining consent ______________________________ Date **XX.09.2018**

Printed name of person obtaining consent ______**Werner Alexander Isop**______ Date **XX.09.2018**

This consent form will be kept by the researcher for at least three years beyond the end of the study.
